# Electroporation increases antitumoral efficacy of the bcl-2 antisense G3139 and chemotherapy in a human melanoma xenograft

**DOI:** 10.1186/1479-5876-9-125

**Published:** 2011-07-28

**Authors:** Enrico P Spugnini, Annamaria Biroccio, Roberta De Mori, Marco Scarsella, Carmen D'Angelo, Alfonso Baldi, Carlo Leonetti

**Affiliations:** 1 S.A.F.U. Department, Regina Elena Cancer Institute, (Via delle Messi d'Oro 156), Rome (00158), Italy; 2 Experimental Chemotherapy Laboratory, Regina Elena Cancer Institute, (Via delle Messi d'Oro 156), Rome, (00158), Italy; 3 Section of Pathology, Department of Biochemistry and Biophysics, Second University of Naples, (Via Costantinopoli 16), Naples, (80138) Italy

## Abstract

**Background:**

Nucleic acids designed to modulate the expression of target proteins remain a promising therapeutic strategy in several diseases, including cancer. However, clinical success is limited by the lack of efficient intracellular delivery. In this study we evaluated whether electroporation could increase the delivery of antisense oligodeoxynucleotides against bcl-2 (G3139) as well as the efficacy of combination chemotherapy in human melanoma xenografts.

**Methods:**

Melanoma-bearing nude mice were treated i.v. with G3139 and/or cisplatin (DDP) followed by the application of trains of electric pulses to tumors. Western blot, immunohistochemistry and real-time PCR were performed to analyze protein and mRNA expression. The effect of electroporation on muscles was determined by histology, while tumor apoptosis and the proliferation index were analyzed by immunohistochemistry. Antisense oligodeoxynucleotides tumor accumulation was measured by FACS and confocal microscopy.

**Results:**

The G3139/Electroporation combined therapy produced a significant inhibition of tumor growth (TWI, more than 50%) accompanied by a marked tumor re-growth delay (TRD, about 20 days). The efficacy of this treatment was due to the higher G3139 uptake in tumor cells which led to a marked down-regulation of bcl-2 protein expression. Moreover, the G3139/EP combination treatment resulted in an enhanced apoptotic index and a decreased proliferation rate of tumors. Finally, an increased tumor response was observed after treatment with the triple combination G3139/DDP/EP, showing a TWI of about 75% and TRD of 30 days.

**Conclusions:**

These results demonstrate that electroporation is an effective strategy to improve the delivery of antisense oligodeoxynucleotides within tumor cells *in vivo *and it may be instrumental in optimizing the response of melanoma to chemotherapy. The high response rate observed in this study suggest to apply this strategy for the treatment of melanoma patients.

## Background

There is currently great interest in the use of oligodeoxynucleotides antisense (ASOs), siRNA and aptamers for the treatment of different diseases, including cancer. Phosphorothioate ASOs are the most widely explored first-generation analogues [1] and preclinical studies have demonstrated that these agents are able to reduce target gene expression and have also shown activity against a wide variety of tumors, both alone and in combination with antineoplastic drugs [2]. Phosphorothioate ASOs have a greater bioavailability than unmodified ASOs, even though they exhibit a short half-life in the blood, low accumulation in tissues and poor intracellular penetration. Therefore, in preclinical and clinical trials, daily intravenous administration or continuous infusion have been used to evaluate the therapeutic efficacy [3-9]. To avoid frequent injections a delivery systems able to protect ASOs from degradation has been used: the encapsulation of ASOs in microspheres or in lipid-based delivery systems was able to improve the delivery of ASOs targeting different oncogenes in several human tumor cells [10-14]. We have previously demonstrated that the biological activity and the therapeutic efficacy of c-myc ASOs is improved when these agents are encapsulated in liposomes [15].

Electroporation therapy (EP) is a treatment modality that uses brief, high-intensity, pulsed electrical currents to enhance the delivery of chemotherapeutic agents, vaccines and genes to cells. *In vitro *studies have shown that the application of high voltage, exponentially-decaying electric pulses to cells in suspension could induce pores in the cell membrane, resulting in cross-membrane flow of material (electroporation, electroinjection) or even in cell fusion when the cells were adjacent [16-19]. This method was initially used to transfect bacterial cells with plasmids, and subsequently exploited to produce monoclonal antibodies through fusion of eukaryotic cells [20]. Later, researchers realized that EP might enhance the transport of drugs and genes through the cytoplasmic membrane by exposing animal cells in culture and plant protoplasts to non-cytotoxic electric pulses [21-23]. Moreover, EP has been proven to be very effective at enhancing the *in vitro *cytotoxicity of anticancer molecules, which in the case of bleomycin, led to an enhancement of 300-700 fold [23].

Only a few clinical trials have been performed in animals and humans over the past ten years, since the first phase I-II EP trial was performed [24]. In these cohorts of patients different voltages, waveforms and delivery modes (i.e. single pulses versus bursts) were tested [24-35].

The results of some studies have shown that electric pulses are capable of driving plasmid into muscle cells resulting in DNA protection from extracellular endonucleases and increased gene expression in rodent and canine models [36,37]. These observations led us to investigate the feasibility of pulse mediated antisense potentiation.

The objective of this study was to evaluate whether electroporation could increase the efficacy of the bcl-2 ASO G3139 on mice bearing human melanoma in combination chemotherapy, in order to identify an innovative approach for antisense delivery to tumors and to increase the response of melanoma to therapy. The rationale for the use of G3139 is based on the relevant role of bcl-2 in melanoma cell survival and on the increased sensitivity of this tumor when it is combined with chemotherapy, as it has been observed in preclinical and clinical studies (5-9).

## Methods

### Tumor cell line and xenografts

The M14 human melanoma line used in this study was derived from melanoma of a patient undergoing surgery at the Regina Elena Cancer Institute (Rome, Italy). The cell line was characterized as previously described [3]. Cells in the exponential phase of *in vitro *growth were injected into the hind leg muscles of mice at 5 × 10^6 ^cells/mouse in 0.2 ml 0.9% NaCl solution. CD-1 male nude, nu/nu mice, 6-8 weeks old and 22-24 g in body weight, purchased from Charles River Laboratories, Calco, Italy, were used. A tumor mass of about 300 mg was evident in all animals on day 6 after implanting the tumor cells. All procedures involving animals and their care were approved by the responsible for the Animal Facility at the Regina Elena Cancer Institute and were conducted in accordance with institutional guidelines, which are in compliance with national (D.L. No. 116, G.U., Suppl. 40, Feb. 18, 1992; Circolare No. 8,G.U., July 1994) and international laws (EEC Council Directive 86/609, OJ L 358. 1, Dec 12, 1987; Guide for the Care and Use of Laboratory Animals, United States National Research Council, 1996).

### Oligodeoxynucleotides and drug

The 18-mer ASO (5'-TCTCCCAGCGTGCGCCAT-3') complementary to the first six codons of bcl-2 mRNA (ASO bcl-2, oblimersen sodium, G3139, GenasenseTM) and the G4243 ODN (FAM- G3139) labeled with 6-fluoroscein on the 5'-T residue, were used (Genta Incorporated, Berkeley Heights, NJ, USA). Clinical-grade DDP (Prontoplatamine) was obtained from Pfizer. DDP dilutions were freshly prepared before each experiment.

### Pulse generator

The Chemopulse [38] is built up by a toroidal core transformer generating a roughly rectangular pulse which is split in two halves that are sequentially driven to obtain a biphasic pulse. The pulses are not singularly produced but are created in bursts of eight, thus reducing the treatment time and the overall patient morbidity. The equipment allows to choose among a broad range of voltages (from 450 to 2450 V) with sequential increases of 200 V and permits to regulate the number of pulses (from 1 to 16) and the pulse duration (50 to 100 μs). The standard train is set to 8 pulses of 50 + 50 μs. The pulse repetition frequency is 1 Hz while the frequency of burst repetition is 1 kHz, resulting in a total burst duration of 7.1 ms. The electrodes used in this study have been extensively previously described [38]. Briefly, modified monolateral compass electrode in steel, bakelite, and plastic with perforated metal plates. Plate dimensions: 22 × 10 × 1 mm, and Vaccine type twin needle array electrode with plastic handle and steel needles. Needle length: 20 mm; array diameter: 20 mm.

### In vivo treatment

To compare the antitumoral activity of electroporation delivered by caliper or needle electrodes, mice were injected i.m. with M14 melanoma cells and treated with the two different modalities, starting from day 6 after implanting tumor cells, when a tumor mass of about 300 mg was evident in all animals. Treatment was carried on for five consecutive days. In particular, sequential bursts of 8 biphasic pulses lasting 50+50 μs were applied to tumor nodules at a voltage of 1300 V/cm for caliper electrodes and 800 V/cm when needle electrodes were adopted [38]. Adherence of the electrodes to the lesion was maximized using an electroconductive gel. To evaluate the antitumoral activity of G3139 and DDP given alone or in combination with EP, M14 melanoma bearing mice were injected i.v. with G3139 at the dose of 0.2 mg/mouse/d for five days or with DDP given i.p. at the dose of 3.3 mg/kg/d for three days and followed, five minutes later, by the delivery of EP to tumors by means of caliper electrodes. The tumor weight was calculated from caliper measurements according to the formula: [(width)^2 ^× length]/2. The antitumor efficacy of the treatments was assessed by the following end-points: a) percent of tumor weight inhibition (TWI%), calculated as [1-(mean tumor weight of treated mice/mean tumor weight of controls)] × 100; b) tumor re-growth delay (TRD), evaluated as the median time (in days) for treated tumors to re-grow after the treatment. Each experimental group included 8 mice.

To evaluate the effects of caliper or needle electrodes treatment on skeletal muscles, experiments have been performed by treating healthy mice with the two different electroporation modality. Histopathological analysis have been performed at the end of treatment on tissue specimens. Each experimental group included three mice and each experiment was repeated three times.

### Western blot analysis

100 mg of mechanically disaggregated control and treated tumors were solubilized in lysis buffer. Briefly, proteins (30 μg) were separated by 10% SDS-PAGE, transferred to nitrocellulose filters, and incubated with monoclonal antibodies specific for human bcl-2 (clone 124, DAKO, Milan, Italy). After stripping, filters were incubated with anti-human β-actin antibody (clone JLA 20; Oncogene Science, Manhasset, NY), and reactivity was detected by enhanced chemiluminescence (Amersham International, Little Chalfont, Buckingamshire, United Kingdom), according to manufacturer's instructions. Results were quantified by scanning densitometry (Bio-Rad G700) of the autoradiography films and normalized to β-actin levels.

### Real-time Polymerase Chain Reaction (PCR)

Total RNA was extracted from tumors by using Trizol reagent following standard protocols (Gibco-BRL, Milano, Italy). Reverse transcription of 0.5 μg of RNA was performed with First-Strand c-DNA Synthesis using SuperScript II random hexamer (Invitrogen, California, USA). The PCR reactions were carried using intercalation of SYBR green following the manufacturer's protocol (Light Cycler DNA Master SYBR Green I, Roche Diagnostics Corp. Indianapolis, USA). Equal amounts of cDNA, as determined by picogreen intercalation (Molecular Probes, Inc., Eugene, OR, USA), were used to quantify the expression of bcl-2 and glyceraldehyde-3-phosphate dehydrogenase (GAPDH) genes. The following primers were used: bcl-2 forward 5'-GTGAACTGGGGGAGGATTGT-3' and reverse 5'-GGAGAAATCAAACAGAGGCC-3'; GAPDH forward 5'-CCAAGGTCATCCATGACAAC-3' and reverse 5'-TTACTCCTTGGAGGCCATGT-3'. Reactions were performed in duplicate from two separate RNA preparations. Relative gene expression was determined as previously described [39].

### Histology, immunohistochemistry and TUNEL

The excised biopsy specimens were fixed in 10% buffered-formalin and paraffin embedded. Sections of 5 μm were stained with haematoxylin-eosin, and haematoxylin-van Gieson. For immunohistochemstry, 5 μm sections were incubated in a microwave oven for 15 minutes in 10 mmol/L, 6.0 pH buffered citrate followed by the immunohistochemical procedure for Ki67 (rabbit polyclonal ab, Santa Cruz Biotechnology Inc., CA, USA) and bcl-2 (mouse monoclonal ab, Dako Carpinteria, CA, USA), diluted 1:100. The conventional avidin-biotin complex procedure was applied according to the manufacturer's protocol (Dako Carpinteria, CA, USA) and then incubated with a secondary antibody. Positive staining was revealed by DAB chromogen, according to the supplier's conditions followed by counterstaining with Mayer Hematoxylin. The slides were cover-slipped with a xylene based, mounting medium and the staining was scored. Negative controls for each tissue section were performed leaving out the primary antibody and positive controls, included in each experiment, consisted of tissues previously shown to express the antigen of interest. TUNEL reaction was performed using the peroxidase-based Apoptag kit (Oncor, Gaithersburg, MD,USA). TUNEL positive cells were detected with DAB and H2O2 according to the supplier's instructions. The experiments were repeated on different sections for each specimen (two to four). For both immunohistochemical markers, one hundred random fields (250X) per section were analyzed (12.5 mm2). Mann-Whitney and Wilcoxon tests were used to assess the relationship between ordinal data. The two-tailed P value was considered significant when ≤ 0.05. SPSS software (version 10.00, SPSS, Chicago, IL, USA) was used for statistical analysis.

### Tumor accumulation of ASO bcl-2 given alone or in combination with electroporation

Mice were injected with ASO bcl-2 labeled with 6-fluorescein on the 5'-T residue (G4243) alone or in combination with EP. Six hours after treatment, mice were euthanized and tumors excised; the tumors were freshly processed to obtain single cell suspension by mechanical disaggregation. The specimens were minced with scissors, washed in PBS and filtered through a 50 μm nylon mesh. Flow cytometric analysis was performed using a FACScan cytofluorimeter (Becton Dickinson, San Jose, CA, USA). The fluorescence signals from 10,000 cells were collected and the results showed in the form of frequency distribution histograms.

In order to detect G3139 labeled in the tumor sections, fresh tumor biopsies were immediately frozen in Optimal Cutting Temperature Compound and microtomic sections were cut with a cryostat. The confocal imaging was performed with a Sarastro Phoibos 1000 confocal laser scanning microscope (Molecular Dynamics, Inc. USA), equipped with an argon ion laser (λ = 488/514 nm). The image processing was performed using the Image Space software (Molecular Dynamics, Inc. USA); the image series were gauss filtered and elaborated independently to obtain look-through projections for FITC images.

Each experimental group included three mice and each experiment was repeated three times.

### Statistical analysis

The statistical differences were determined using the Student's t test, assuming unequal variances. Differences were considered significant at *P *values < .05 (two sided).

## Results

### Antitumor activity of EP given by caliper or needle electrodes

**Figure 1:**
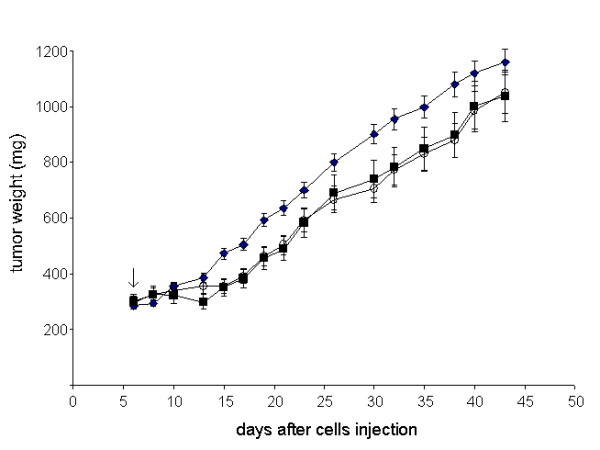
**Antitumor activity of EP given by caliper or needle electrodes**. Mice were implanted i.m. with M14 melanoma cells and after six days, treated for five consecutive days according to the following schedules:(black diamond), untreated; (black square), caliper electrodes; (open circle), needle electrodes. Sequential bursts of 8 biphasic pulses lasting 50+50 μs were applied to tumor nodules. Mean tumor weight in mg ± s.d. are shown. Arrow indicates the start of treatments. Each experimental group included 8 mice.

**Figure 2:**
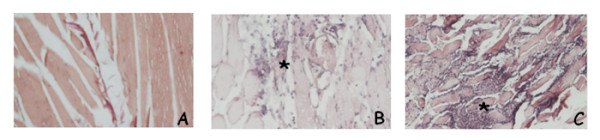
**Histopathological analysis of skeletal muscle of mice untreated or treated by caliper or needle electrodes**. Panel A: cross-section of normal skeletal muscle from an untreated mouse (original magnification ×20); Panel B: cross-section of skeletal muscle from a mouse treated by caliper electrodes showing a focus of mild mononuclear inflammation, indicated by an asterisk (original magnification ×20); Panel C: cross-section of skeletal muscle from a mouse treated by needle electrodes displaying a more severe mononuclear inflammation with necrosis and phagocytosis of muscle fibers, indicated by an asterisk (original magnification ×20).

To choose the more suitable method of EP, mice bearing M14 human melanoma were treated with electric pulses, delivered to tumor nodules, by two different electrodes: caliper or needle. As reported in Figure 1, no differences in terms of reduction of tumor growth were observed between the two treatments. In fact, a maximum of 20% TWI was elicited by the two different modalities of treatment. Interestingly, the histopathological analysis of the effects of EP given by caliper or needle electrodes on skeletal muscles of healthy mice showed that caliper electrodes caused only mild interstitial myositis, while needle electrodes caused more severe myositis with necrosis and phagocytosis of the muscle fibers, and fibrosis (Figure 2). Since we inserted the needle within the posterior muscles of the thigh, a more severe damage was observed at the insertion points. Similar results were obtained in three independent experiments. Based on these observations the following experiments were performed by using caliper electrodes.

### Antitumor efficacy of G3139 alone or in combination with EP

**Figure 3:**
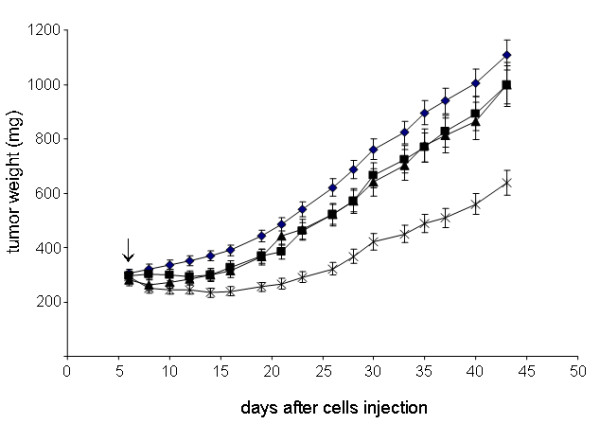
**Effect of G3139 alone or in combination with EP on the growth of M14 tumor cells implanted in mice**. (black diamond), untreated; (black square), EP alone; (black triangle),G3139 alone; (asterisk), G3139 and EP. Mice were injected i.v. with G3139 at the dose of 0.2 mg/mouse/day and followed, five minutes later, by the delivery of electric pulses to tumors by means of caliper electrodes. Treatments were repeated for five consecutive days. Mean tumor weights in mg ± s.d. are shown. Arrow indicates the start of treatment. Each experimental group included 8 mice.

As shown in Figure 3, treatment with G3139 or EP alone produced a slight reduction of tumor growth (about 20% TWI); conversely, the association with EP was able to increase the efficacy of the antisense. In fact, a marked inhibition of the tumor growth, evaluated at the nadir of the effect, was observed (greater than 50%). This effect favorably compares untreated mice (P = 0.007), with mice treated with G3139 or EP (*P *= 0.001). More interestingly, a stabilization of tumor growth was observed in mice treated with the combination therapy lasting for more than 20 days, after which tumor relapse was observed. In mice treated with EP or G3139 alone, a stabilization of tumors was also observed, but only for a short time (about 4 days).

### Bcl-2 down-regulation

**Figure 4:**
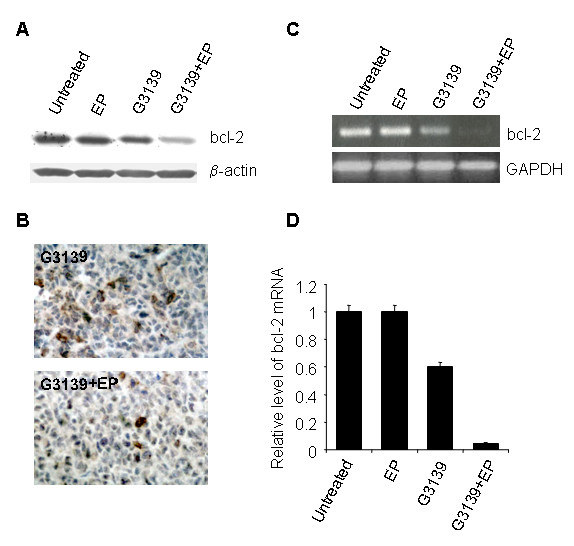
**Effect of G3139 alone or in combination with EP on protein and mRNA expression in M14 tumors**. Tumors were excised from mice on day 4 after the end of treatment. Total protein or RNA was obtained from a pool of three different tumors. Treatments were as shown. Panel A: Western blot analysis of bcl-2 protein quantified and normalized to β-actin protein amount. Panel B: representative immunohistochemical analysis of bcl-2 protein in tumor sections (original magnification, × 40). Panel C: Real-time PCR of bcl-2 mRNA expression. GAPDH mRNA expression was used as internal control. Panel D: Relative level of bcl-2 gene expression calculated as a ratio of the quantity of bcl-2 and GAPDH PCR products.

To determine whether the enhanced anti-tumor activity elicited by the treatment with G3139 followed by EP was correlated to differences in intra-tumoral bcl-2 protein levels, Western blot analysis was performed in tumors excised from all the groups of mice (Figure 4A). Densitometric analysis showed that on day 4 after the end of treatment, only a minimal reduction of bcl-2 protein expression (<10%) was observed in tumors from mice treated with electric pulses, while the effect on bcl-2 expression was more pronounced (~30% reduction), following the administration of G3139. The treatment with G3139/EP combination produced more than a 70% reduction of the bcl-2 protein expression. Similarly, immunohistochemical staining corroborated this level of reduction for bcl-2 protein in tumors treated with the G3139/EP combination compared to the treatment with G3139 alone (Figure 4B). Finally, quantitative analysis of m-RNA of bcl-2 by RT-PCR confirmed the highest efficacy of the combination in reducing the expression of the targeted gene (Figure 4C and 4D).

### Apoptosis and proliferation index in tumors after treatment with G3139 alone or in combination with EP

**Figure 5:**
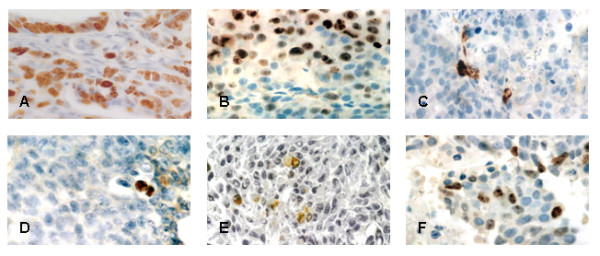
**Proliferation index and apoptosis in M14 tumors treated with G3139 alone or in combination with EP**. Tumors were excised from mice on day 4 after the end of treatment. Sections shown are as follows: Ki67 expression in tumors from animals untreated (panel A), treated with G3139 alone (panel B) or treated with G3139 in combination with EP (panel C); TUNEL staining in tumors from mice untreated (panel D), treated with G3139 alone (panel E), or treated by the combination (panel F). Original magnification, × 40.

In order to ascertain if the marked antitumor efficacy observed in mice treated with EP in combination with G3139 was due to reduced cell proliferation and/or enhanced apoptosis, Ki67 and TUNEL scores were performed at the end of the treatment. Statistical analysis of the scores obtained revealed that the proliferation index was significantly lower in tumors of mice receiving the combination treatment compared to the group treated with G3139 alone (15% *vs *35%, p = 0.002). Accordingly, the apoptotic index was significantly higher in the former group (15 ± 3 *vs *7 ± 2, P = 0.002). Representative findings of these analyses are reported in Figure 5. Proliferation and apoptotic index in EP treated tumors was not significantly different compared to untreated tumors (data not shown).

### Tumor accumulation of G3139 alone or in combination with EP

**Figure 6:**
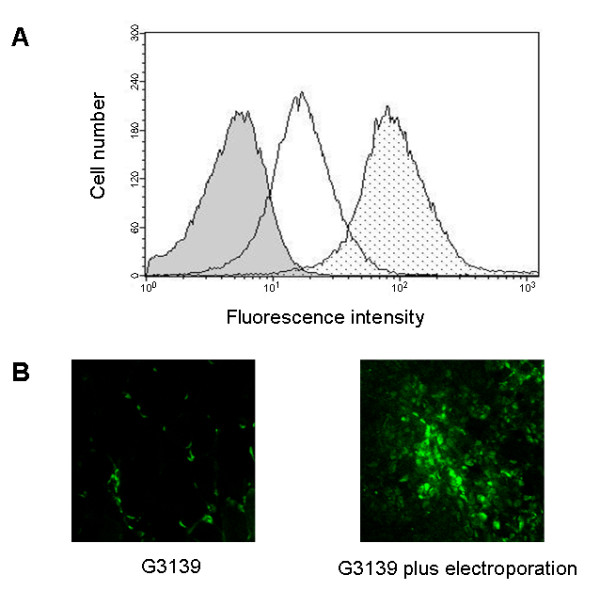
**G3139 tumor accumulation after treatment alone or combined with EP**. Mice were treated with G3139 labeled with 6-fluoroscein and six hours after treatment the tumors were excised. Results are as follows: Panel A, flow cytometric analysis of G3139 content in tumor cells from mice treated with EP (grey area) or G3139 (blank area) alone or in combination (dotted area). Panel B: representative sections of tumors from mice treated with G3139 alone (left) or in combination with EP (right). Original magnification × 20.

Mice bearing M14 tumors were injected i.v. with a single dose of G3139, labeled with fluorescein and then randomized in two groups, those receiving or not receiving electric pulses. FACS analysis of the G3139 content in cells from the differently treated tumors is shown in Figure 6A. Application of EP was able to markedly increase the intratumoral concentration of G3139 compared to tumors excised from mice treated with oligos alone.

Consistently, the analysis of G3139 distribution performed in tumor sections by confocal microscopy showed a higher number of tumor cells incorporating the oligos in mice treated by the application of EP (Figure 6B). Similar results were obtained in three independent experiments.

### Antitumor activity of G3139 and DDP in combination with EP

Based on the results reported above, showing that the biological activity of G3139 is increased when EP is applied to tumors, and with the aim to identify a more effective antimelanoma therapy, we evaluated the therapeutic efficacy of a multicomponent strategy based on the use of EP, G3139 and DDP, a drug currently employed in clinical management of melanoma patients. As reported in Table 1, the combination of G3139 and DDP produced a marked antitumoral effect with a TWI of more than 50% and 20 days of TRD being observed. Interestingly, the addition of EP produced a more relevant antitumoral efficacy, reaching an inhibition of tumor growth of about 75%: significantly different compared to the G3139 and DDP combination (P = 0.007), G3139 and EP combination (p = 0.007) or the untreated and the other treated groups (P < 0.001). Moreover, the triple combination produced a more sustained regression of tumor growth than the other groups, lasting for 30 days.

**Table 1:** Antitumor efficacy of G3139 alone or in combination with chemotherapy and EP on M14 melanoma-bearing mice

Groups (treatment days)^#^	TWI* (%)	TRD^§^ (days)
a) EP (days 6-13)	25	8
b) G3139 (days 6-10)	26	9
c) G3139/EP (days 6-10)	51	18
d) DDP (days 6-8)	31	10
e) DDP/EP (days 6-8)	43	15
f) G3139/DDP (days 6-13)	52	20
g) G3139/DDP/EP (days 6-13)	74	30

^# ^Mice were injected i.v. with G3139 at the dose of 0.2 mg/mouse/day for five days and/or with DDP given i.p at 3.3 mg/kg/days for three days and followed, five minutes later, by the delivery of EP to tumors by means of caliper electrodes. Treatment started at day 6 after tumor cell injection and was continued for the days indicated in parentheses. Each experimental group included 8 mice.

*Tumor weight inhibition was calculated at the nadir of the effect comparing treated versus untreated groups.

^§^Tumor re-growth delay was evaluated as the median time (in days) for treated tumors to re-grow after the treatment.

## Discussion

Melanoma has become an increasing source of concern due to its growing incidence among Caucasians. While early stage melanomas (melanoma in situ, Breslow thickness II- III A) can be treated with surgical excision alone, advanced melanoma has a poor prognosis [40]. The role of radiation therapy is confined to the treatment of loco-regional disease, especially in those areas where aggressive surgery is not feasible, such as head and neck melanoma. Adjuvant therapies of metastatic melanoma have been unrewarding with a median survival of 6 to 7.5 months and a 5 year survival of 6% [41]. Treatment options include chemotherapy with dacarbazine, platinum analogues, chloronitrosureas, vindesine, temozolomide, taxanes, immunotherapy with interferon, interleukin and BCG [41,42]. However there is no proof that systemic treatment prolongs patient survival.

Due to the intrinsic chemoresistance of malignant melanoma, novel strategies are currently being investigated in order to increase tumor control, such as ASOs. These agents have shown convincing *in vitro *reduction of target expression and promising activity against a wide variety of tumors in preclinical studies [2]; moreover, phase III trials incorporating G3139 have recently been completed in different advanced cancers, including melanoma [9].

One of the approaches recently adopted by some investigators involves attacking melanomas with the association of chemotherapy and square electric pulses (electrochemotherapy). The first report by Sersa et al. [43] suggested a total response of 78% in ten patients with multiple metastatic nodules. Since then, these results have also been confirmed by other clinical investigations [44,45]. Furthermore, electrochemotherapy has been used with a certain degree of success to palliate patients with multiple cutaneous nodules [46].

Our experimental protocol has been designed on the basis of recent results obtained in a spontaneous *in vivo *model of oral melanoma in dogs [33]. Canine patients were treated with trains of biphasic electric pulses coupled with loco-regional chemotherapy with bleomycin leading to enhanced local control and prolonged survival. Electrochemotherapy is still mostly used for the treatment of cutaneous and subcutaneous located neoplasms [47]. However, several groups are currently working on the development of equipment and electrodes specifically tailored for the treatment of visceral neoplasms, like those for the irreversible electroporation of such lesions [48-50]. Another aspect of electroporation that should be emphasized is that it mainly enhances the penetration of lipophobic molecules such as bleomycin, methotrexate (up to 700 fold) while the uptake of cisplatin is improved by a 4 fold factor. However, this mechanism leads to the apoptotic death of tumor cells resulting in a high tumoricidal effect with good cosmetic results (minimal scar tissue formation in the treated patients). A final advantage of electrochemotherapy is the possible modulation of the immune system, probably through the uncovering of cancer antigens, as suggested by studies showing a synergy with interleukin 2 or 12 [51,52]. Furthermore, our investigations on companion animals with spontaneous cancer evidenced a clonal selection of the neoplasms treated with electrochemotherapy and longer survival in dogs with melanoma that developed vitiligo-like lesions at the tumor site after electroporation [53]. The ability to induce such response through electroporation would be of paramount importance for melanoma patients with advanced disease.

The data presented in this study demonstrate that proper electric waveforms can enhance the delivery and activity of ASOs within solid tumors. This, in turn, induces a better antitumor efficacy than that obtained by the ASOs as single agents. In this regard, the improved delivery is clearly evidenced by the increased accumulation of fluorescein-labeled ASOs in tumor lysates from mice treated with EP, as observed by FACS analysis. In addition, confocal microscope analysis of tumor sections from animals treated with the combined modality, confirms a higher uptake of ASOs and shows the proper intracellular localization of the fluorescent ASOs.

Consistently, we observed that tumors treated with the combination of G3139 and EP display a lower expression of bcl-2 both at the transcriptional and translational levels, as evidenced by quantitative RT-PCR and Western blot analyses, than that observed with the treatment with G3139 alone. Bcl-2 protein is expressed in most tissue but it is overexpressed in tumors [54]. The ability of G3139 to distribute in organs and to decrease Bcl-2 protein level has been observed in clinical studies. In fact, after i.v. administration G3139 was detected in plasma, kidneys and at low levels in lung, heart and muscle [55]. Moreover, a phase I study showed that G3139 reduced the Bcl-2 protein levels in normal peripheral blood mononuclear cells [56].

The lowered bcl-2 expression had a direct influence on cell proliferation and apoptosis as demonstrated by Ki-67 and TUNEL analysis. Indeed, the differences found in the apoptotic scores are significant, especially if we consider that we are working with an *in vivo *system. An observed doubled apoptotic index in the tumors treated with G3139/EP combination compared to tumors treated with G3139, justifies the different biological behavior of the tumors. These biological effects, ultimately resulted in marked tumor reduction, as shown by the tumor growth curves. It is important to outline that additional "non-specific" mechanisms may contribute to the antitumoral effects of G3139 which are likely to depend on the presence of the "bis-CpG" motif in their sequence [57].

In addition to bcl-2 ASO, EP also improved the antitumor efficacy of ASO targeting c-myc mRNA (data not shown). In fact, we observed that treatment of melanoma M14 bearing mice with EP and ASO c-myc (INX-3280) in combination, resulted in a marked inhibition (46%) of tumor weight, a significant increase compared to mice treated with INX-3280 (33%, P = 0.028) or with EP alone (28%, P = 0.002).

The ability of EP to increase accumulation of antineoplastic drugs injected systemically has previously been reported by Cemazar et al [58]. These authors showed an increased amount of DDP in cells obtained from tumors of mice treated with electrochemotherapy, as a result of increased permeability of the tumor cell membranes. The modification of tumor blood flow observed after the application of EP [59,60] may also account for the higher concentration of drugs in the tumor and for the better antitumor effectiveness of chemotherapy. Interestingly, in accordance with these observations, our result demonstrate that the integration of DDP with bcl-2 ASO and EP produced an impressive antitumor efficacy on this melanoma model, suggesting a possible translational application of this therapeutic strategy.

## Conclusions

These results demonstrate that pulse mediated ASOs delivery seems to be a promising approach to cutaneous melanoma, especially in view of the high tolerability and low toxicity evidenced in our experimental data. Of note, the adoption of caliper electrodes greatly minimize the local side effects to normal tissues adjacent to neoplastic lesions, that are limited to mild and self-limiting myositis.

To the best of our knowledge, this is the first report of increased vehicolation by electroporation of ASOs in a tumor xenograft. Our results highlight that electric pulses of appropriate waveform can be instrumental in increasing the delivery of antisense molecules to tumors. Further studies are warranted to establish the adoption of this treatment, possibly in combination with chemotherapeutic drugs, in more clinically oriented settings.

## Abbreviations

(ASOs): Oligodeoxynucleotides; (EP): electroporation; (TWI%): percent tumor weight inhibition; (TRD): tumor re-growth delay; (PCR): Real-time Polymerase Chain Reaction; (DDP): cisplatin.

## Competing interests

The authors declare that they have no competing interests.

## Authors' contributions

AB performed statistical analysis and made substantial contribution to the interpretation of data. RDM and MS performed the *in vivo *antitumor efficacy studies. CD carried out flow cytometric, western blot and PCR analysis. AB performed histological, immunohistochemistry and confocal microscopy studies. EPS and CL conceived and designed the study, writed and guided the editing of the manuscript. All authors read and approved the final manuscript.

## References

[B1] Eckstein F Phosphorothioate oligodeoxynucleotides: what is their origin and what is unique about them? Antisense Nucleic Acid Drug Dev 2000 10 117 121 10.1089/oli.1.2000.10.117 10805163

[B2] Gleave ME Monia BP Antisense therapy for cancer Nat Rev Cancer 2005 5 468 479 10.1038/nrc1631 15905854

[B3] Leonetti C D'Agnano I Lozupone F Valentini A Geiser T Zon G Calabretta B Citro GC Zupi G Antitumor effect of c-myc antisense phosphorothioate oligodeoxynucleotides on human melanoma cells in vitro and and in mice J Natl Cancer Inst 1996 88 419 429 10.1093/jnci/88.7.419 8618233

[B4] Leonetti C Biroccio A Candiloro A Citro G Fornari C Mottolese M Del Bufalo D Zupi G Increase of cisplatin sensitivity by c-myc antisense oligodeoxynucleotides in a human metastatic melanoma inherently resistant to cisplatin Clin Cancer Res 1999 5 2588 2595 10499637

[B5] Zupi G Scarsella M Semple SC Mottolese M Natali PG Leonetti C Antitumor efficacy of bcl-2 and c-myc antisense oligonucleotides in combination with cisplatin in human melanoma xenografts: relevance of administration sequence Clinical Cancer Res 2005 11 1990 1995 10.1158/1078-0432.CCR-04-1284 15756025

[B6] Leonetti C Biroccio A D'Angelo C Semple SC Scarsella M Zupi G Therapeutic integration of c-myc and bcl-2 antisense molecules with docetaxel in a preclinical model of hormone-refractory prostate cancer Prostate 2007 67 1475 1485 10.1002/pros.20636 17654511

[B7] Jansen B Schlagbauer-Wadl H Brown BD Bryan RN van Elsas A Müller M Wolff K Eichler HG Pehamberger H bcl-2 antisense therapy chemosensitizes human melanoma in SCID mice Nat Med 1998 4 232 234 10.1038/nm0298-232 9461199

[B8] Jansen B Wacheck V Heere-Ress E Schlagbauer-Wadl H Hoeller C Lucas T Hoermann M Hollenstein U Wolff K Pehamberger H Chemosensitisation of malignant melanoma by BCL2 antisense therapy Lancet 2000 356 1728 1733 10.1016/S0140-6736(00)03207-4 11095261

[B9] Bedikian AY Millward M Pehamberger H Conry R Gore M Trefzer U Pavlick AC DeConti R Hersh EM Hersey P Kirkwood JM Haluska FG Oblimersen Melanoma Study Group Bcl-2 antisense (oblimersen sodium) plus dacarbazine in patients with advanced melanoma J Clin Oncol 2006 24 4738 4745 10.1200/JCO.2006.06.0483 16966688

[B10] Putney SD Brown J Cucco C Lee R Skorski T Leonetti C Geiser T Calabretta B Zupi G Zon G Enhanced anti-tumor effects with microencapsulated c-myc antisense oligonucleotide Antisense Nucleic Acid Drug Dev 1999 9 451 458 10.1089/oli.1.1999.9.451 10555152

[B11] Gokhale PC Soldatenkov V Wang F-H Rahman A Dritschilo A Kasid U Antisense raf oligodeoxynucleotide is protected by liposomal encapsulation and inhibit Raf-1 protein expression in vitro and in vivo: Implications for gene therapy of radioresistant cancer Gene Ther 1997 4 1289 1299 10.1038/sj.gt.3300543 9472552

[B12] Gokhale PC McRae D Monia BP Bagg A Rahman A Dritschilo A Kasid U Antisense raf oligodeoxyribonucleotide is a radiosensitizer in vivo Antisense Nucleic Acid Drug Dev 1999 9 191 201 10.1089/oli.1.1999.9.191 10355825

[B13] Pagnan G Stuart DD Pastorino F Raffaghello L Montaldo PG Allen TM Calabretta B Ponzoni M Delivery of c-myb antisense oligodeoxynucleotides to human neuroblastoma cells via disialoganglioside GD2-targeted immunoliposomes: antitumor effects J Natl Cancer Inst 2000 92 253 261 10.1093/jnci/92.3.253 10655443

[B14] Stuart DD Kao GY Allen TM A novel, long-circulating, and functional liposomal formulation of antisense oligodeoxynucleotides targeted against MDR1 Cancer Gene Ther 2000 7 466 475 10.1038/sj.cgt.7700145 10766353

[B15] Leonetti C Biroccio A Benassi B Stringaro A Stoppacciaro A Semple SC Zupi G Encapsulation of c-myc antisense oligodeoxynucleotides in lipid particles improves antitumoral efficacy in vivo in a human melanoma line Cancer Gene Ther 2001 8 459 468 10.1038/sj.cgt.7700326 11498766

[B16] Senda M Takeda J Abe S Nakamura T Induction of cell fusion of plant protoplasts by electrical stimulation Plant Cell Physiol 1979 20 1441 1443

[B17] Zimmermann U Scheurich P High frequency fusion of plant protoplasts by electric fields Planta 1981 151 26 32 10.1007/BF00384233 24301666

[B18] Sugar IP Neumann E Stochastic model for electric field-induced membrane pores Biophysical Chemistry 1984 19 211 225 10.1016/0301-4622(84)87003-9 6722274

[B19] Mir LM Banoun H Paoletti C Introduction of definite amounts of nonpermanent molecules into living cells after electropermeabilization: direct access to cytosol Exp Cell Res 1988 175 15 25 10.1016/0014-4827(88)90251-0 3345798

[B20] Lo MMS Tsong TY Conrad MK Strittmatter SM Hester LD Snyder SH Monoclonal antibody production by receptor-mediated electrically induced cell fusion Nature 1984 31 792 794 10.1038/310792a0 6088990

[B21] Orlowski S Belehradec J Paoletti C Mir LM Transient electropermeabilization of cells in culture. Increase of the cytotoxicity of anticancer drugs Biochem Pharmacol 1988 37 4727 4733 10.1016/0006-2952(88)90344-9 2462423

[B22] Fromm M Taylor LP Walbot V Expression of genes transferred into monocot and dicot plant cells by electroporation Proc Nat Acad Sci USA 1985 82 5824 5828 10.1073/pnas.82.17.5824 3862099 PMC390645

[B23] Poddevin B Orlowski S Belehradek J Jr Mir LM Very high cytotoxicity of bleomycin introduced into the cytosol of cells in culture Biochem Pharmacol 1991 42 Suppl 67 75 10.1016/0006-2952(91)90394-k 1722669

[B24] Belehradek M Domenge C Luboinski B Orlowski S Belehradek J Jr Mir LM Electrochemotherapy, a new antitumor treatment. First clinical phase I-II trial Cancer 1993 72 3694 3700 10.1002/1097-0142(19931215)72:12<3694::AID-CNCR2820721222>3.0.CO;2-2 7504576

[B25] Rudolf Z Stabuc B Cemazar M Miklavcic D Vodovnik L Sersa G Electrochemotherapy with bleomycin. The first clinical experience in malignant melanoma patients Radiol Oncol 1995 29 229 235

[B26] Heller R Jaroszeski M Glass FL Messina JL Rapaport DP DeConti RC Fenske NA Gilbert RA Mir LM Reintgen DS Phase I/II trial for the management of cutaneous and subcutaneous tumors using electrochemotherapy Cancer 1996 77 964 971 10.1002/(SICI)1097-0142(19960301)77:5<964::AID-CNCR24>3.0.CO;2-0 8608491

[B27] Daskalov I Mudrov N Peycheva E Exploring new instrumentation parameters for electrochemotherapy IEEE Eng Med Biol Mag 1999 18 62 66 10.1109/51.740982 9934602

[B28] Spugnini EP Dotsinsky I Mudrov N Citro G D'Avino A Baldi A Biphasic pulses enhance bleomycin efficacy in a spontaneous canine genital tumor model of chemoresistance: Sticker sarcoma J Exp Clin Cancer Res 2008 27 58 10.1186/1756-9966-27-58 18980687 PMC2596090

[B29] Spugnini EP Porrello A Potentiation of chemotherapy in companion animals with spontaneous large neoplasms by application of biphasic electric pulses J Exp Clin Cancer Res 2003 22 571 580 15053299

[B30] Spugnini EP Vincenzi B Baldi F Citro G Baldi A Adjuvant electrochemotherapy for the treatment of incompletely resected canine mast cell tumors Anticancer Res 2006 26 4585 4589 17201181

[B31] Spugnini EP Baldi A Vincenzi B Bongiorni F Bellelli C Porrello A Intraoperative versus postoperative electrochemotherapy in soft tissue sarcomas: a preliminary study in a spontaneous feline model Cancer Chemother Pharmacol 2007 59 375 381 16807731 10.1007/s00280-006-0281-y

[B32] Spugnini EP Vincenzi B Citro G Santini D Dotsinsky I Mudrov N Montesarchio V Laieta MT Esposito V Baldi A Adjuvant electrochemotherapy for the treatment of incompletely excised spontaneous canine sarcomas In Vivo 2007 21 819 822 18019417

[B33] Spugnini EP Baldi F Mellone P Feroce F D'Avino A Bonetto F Vincenzi B Citro G Baldi A Patterns of tumor response in canine and feline cancer patients treated with electrochemotherapy: preclinical data for the standardization of this treatment in pets and humans J Transl Med 2007 5 48 10.1186/1479-5876-5-48 17910745 PMC2082020

[B34] Spugnini EP Citro G Mellone P Dotsinsky I Mudrov N Baldi A Electrochemotherapy for localized lymphoma: a preliminary study in companion animals J Exp Clin Cancer Res 2007 26 343 346 17987793

[B35] Peycheva E Daskalov I Tsoneva I Electrochemotherapy of Mycosis fungoides by interferon-alpha Bioelectrochemistry 2007 70 283 286 10.1016/j.bioelechem.2006.10.006 17150416

[B36] Mennuni C Calvaruso F Zampaglione I Rizzuto G Rinaudo D Dammassa E Ciliberto G Fattori E La Monica N Hyaluronidase increases electrogene transfer efficiency in skeletal muscle Hum Gene Ther 2002 13 355 365 10.1089/10430340252792495 11860703

[B37] Zaharoff DA Barr RC Yuan F Electromobility of plasmid DNA in tumor tissues during electric field-mediated gene delivery Gene Ther 2002 9 1286 1290 10.1038/sj.gt.3301799 12224011

[B38] Spugnini EP Citro G Porrello A Rational design of new electrodes for electrochemotherapy J Exp Clin Cancer Res 2005 24 245 254 16110758

[B39] Livak KJ Schmittgen TD Analysis of relative gene expression data using real-time quantitative PCR and the 2(-Delta Delta CT) Method Methods 2001 25 402 408 10.1006/meth.2001.1262 11846609

[B40] Dummer R Hauschild A Jost L ESMO Guidelines Working Group Cutaneous malignant melanoma: ESMO clinical recommendations for diagnosis, treatment and follow-up Ann Oncol 2008 19 Suppl 2 ii86 ii88 18456782 10.1093/annonc/mdn100

[B41] Mouawad R Sebert M Michels J Bloch J Spano JP Khayat D Treatment for metastatic malignant melanoma: old drugs and new strategies Crit Rev Oncol Hematol 2010 74 27 39 10.1016/j.critrevonc.2009.08.005 19781957

[B42] Argawala SS Neuberg D Park Y Kirkwood JM Mature results of a phase III, randomized trial of bacillus Calmette-Guerin (BCG) versus observation and BCG plus dacarbazine versus BCG in the adjuvant therapy of American Joint Committee on Cancer stage I-III melanoma (E1673): a trial of the Eastern Oncology Group Cancer 2004 100 1692 1698 10.1002/cncr.20166 15073858

[B43] Sersa G Stabuc B Cemazar M Miklavcic D Rudolf Z Electrochemotherapy with cisplatin: clinical experience in malignant melanoma patients Clin Cancer Res 2000 6 863 867 10741708

[B44] Rols MP Bachaud JM Giraud P Chevreau C Roche H Teissie J Electrochemotherapy of cutaneous metastases in malignant melanoma Melanoma Res 2000 10 468 474 10.1097/00008390-200010000-00009 11095408

[B45] Byrne CM Thompson JF Johnston H Hersey P Quinn MJ Michael Hughes T McCarthy WH Treatment of metastatic melanoma using electroporation therapy with bleomycin (electrochemotherapy) Melanoma Res 2005 15 45 51 10.1097/00008390-200502000-00008 15714120

[B46] Gehl J Geertsen PF Efficient palliation of haemorrhaging malignant melanoma skin metastases by electrochemotherapy Melanoma Res 2000 10 1 5 10.1097/00008390-200012000-00011 11198481

[B47] Landström FJ Nilsson CO Crafoord S Reizenstein JA Adamsson GB Löfgren LA Electroporation therapy of skin cancer in the head and neck area Dermatol Surg 2010 36 1245 1250 10.1111/j.1524-4725.2010.01617.x 20666812

[B48] Pech M Janitzky A Wendler JJ Strang C Blaschke S Dudeck O Ricke J Liehr UB Irreversible electroporation of renal cell carcinoma: A first-in-man Phase I clinical study Cardiovasc Intervent Radiol 2011 34 132 138 10.1007/s00270-010-9964-1 20711837

[B49] Lee EW Chen C Prieto VE Dry SM Loh CT Kee ST Advanced hepatic ablation technique for creating complete cell death: irreversible electroporation Radiology 2010 255 426 433 10.1148/radiol.10090337 20413755

[B50] Neal RE Singh R Hatcher HC Kock ND Torti SV Davalos RV Treatment of breast cancer through the application of irreversible electroporation using a novel minimally invasive single needle electrode Breast Cancer Res Treat 2010 123 295 301 10.1007/s10549-010-0803-5 20191380 PMC3021965

[B51] Reed SD Fulmer A Buckholz J Zhang B Cutrera J Shiomitsu K Li S Bleomycin/interleukin-12 electrochemogenetherapy for treating naturally occurring spontaneous neoplasms in dogs Cancer Gene Ther 2010 17 571 578 10.1038/cgt.2010.13 20414325

[B52] Horton HM Lalor PA Rolland AP IL-2 plasmid electroporation: from preclinical studies to phase I clinical trial Methods Mol Biol 2008 423 361 372 10.1007/978-1-59745-194-9_28 18370214

[B53] Spugnini EP Dragonetti E Vincenzi B Onori N Citro G Baldi A Pulse mediated chemotherapy enhances local control and survival in a spontaneous canine model of primary mucosal melanoma Melanoma Res 2006 16 23 27 10.1097/01.cmr.0000195702.73192.a0 16432452

[B54] Reed JC Bcl-2-family proteins and hematologic malignancies: history and future prospects Blood 2008 111 3322 3330 10.1182/blood-2007-09-078162 18362212 PMC2275002

[B55] Lopes de Menezes DE Mayer LD Pharmacokinetics of Bcl-2 antisense oligonucleotide (G3139) combined with doxorubicin in SCID mice bearing human breast cancer solid tumor xenografts Cancer Chemother Pharmacol 2002 49 57 68 10.1007/s00280-001-0385-3 11855753

[B56] Mita MM Ochoa L Rowinsky EK Kuhn J Schwartz G Hammond LA Patnaik A Yeh IT Izbicka E Berg K Tolcher AW A phase I, pharmacokinetic and biologic correlative study of oblimersen sodium (Genasense, G3139) and irinotecan in patients with metastatic colorectal cancer Ann Oncol 2006 17 313 321 16322117 10.1093/annonc/mdj067

[B57] Lai JC Benimetskaya L Santella RM Wang Q Miller PS Stein CA ODN bcl-2 (oblimersen) may inhibit prostate cancer cell growth in a partially bis-CpG-dependent non-antisense manner Mol Cancer Ther 2003 2 1031 1043 14578468

[B58] Cemazar M Miklavcic D Scancar J Dolzan V Golouh R Sersa G Increased platinum accumulation in SA-1 tumour cells after in vivo electrochemotherapy with cisplatin Br J Cancer 1999 79 1386 1391 10.1038/sj.bjc.6690222 10188880 PMC2374264

[B59] Sersa G Cemazar M Miklavcic D Chaplin DJ Tumor blood flow modifying effect of electrochemotherapy with bleomycin Anticancer Research 1999 19 4017 4022 10628347

[B60] Sersa G Jarm T Kotnik T Coer A Podkrajsek M Sentjurc M Miklavcic D Kadivec M Kranjc S Secerov A Cemazar M Vascular disrupting action of electroporation and electrochemotherapy with bleomycin in murine sarcoma Br J Cancer 2008 98 388 398 10.1038/sj.bjc.6604168 18182988 PMC2361464

